# Supplementary material for: The Influence of Cellulose Ethers on the Physico-Chemical Properties, Structure and Lipid Digestibility of Animal Fat Emulsions Stabilized by Soy Protein
Source: Foods. 2022 Mar 2;11(5):738. doi: 10.3390/foods11050738 (PMC8909280; doi:10.3390/foods11050738)
Supplement: Supplementary file 1 [file foods-11-00738-s001.zip › foods-1610338-supplementary.pdf]

foods

MDPI

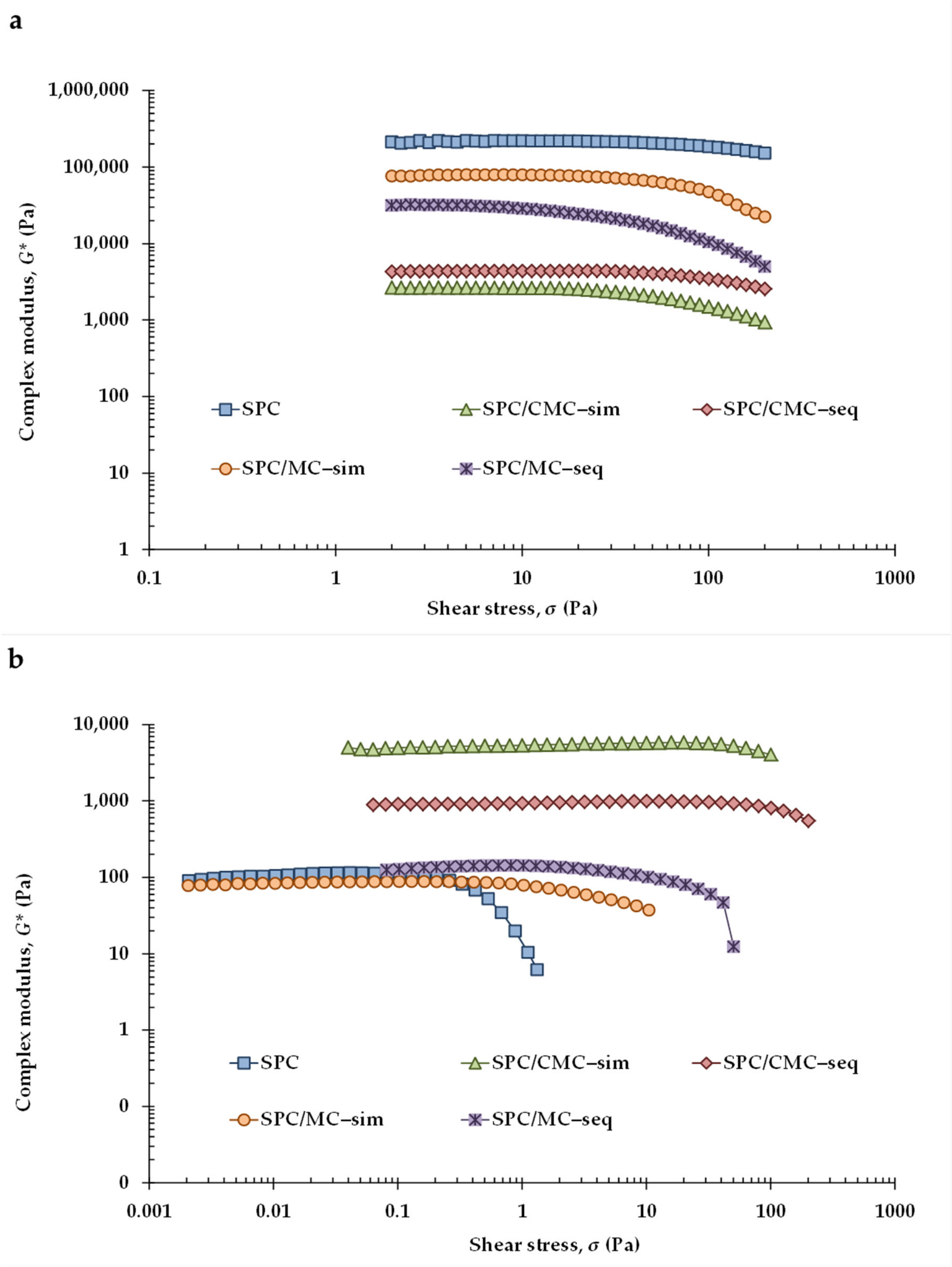

Figure S1. Complex modulus (G^*) as a function of the applied shear stress for the emulsions: (**a**) at 5 °C; (**b**) at 37 °C.

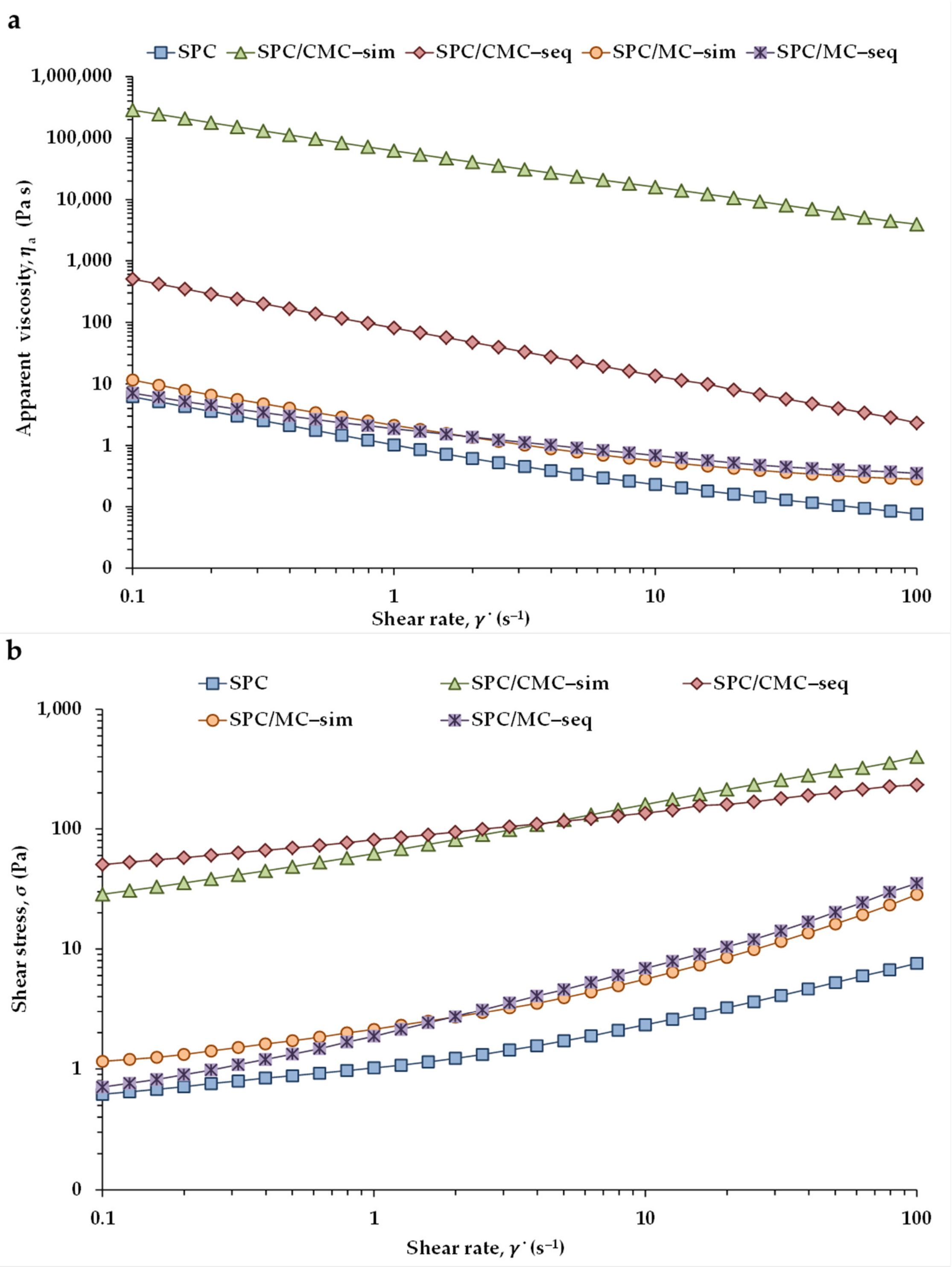

Figure S2. Apparent viscosity (η_a) as a function of the time derived from three step shear rate tests for the emulsions at 37 °C.

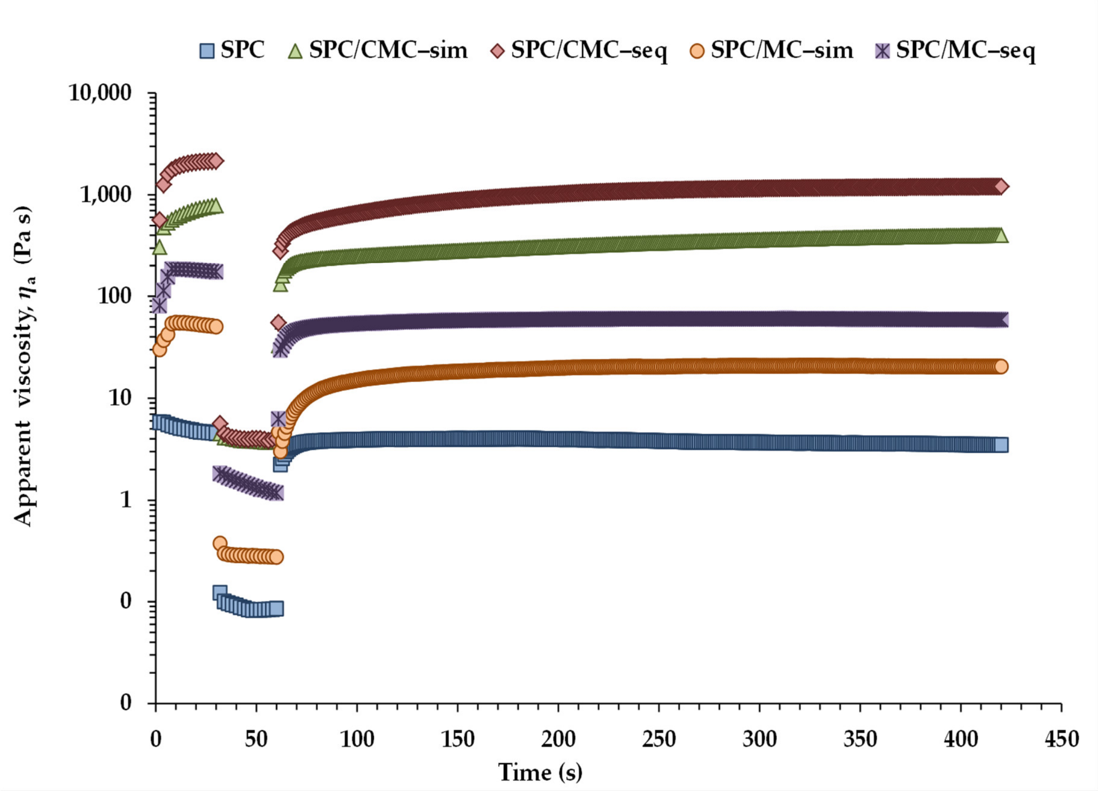

Figure S3. Apparent viscosity (η_a) as a function of the time derived from three step shear rate tests for the emulsions at 37 °C.
